# Randomized Trial Examining Effects of Animal Assisted Intervention and Stress Related Symptoms on College Students’ Learning and Study Skills

**DOI:** 10.3390/ijerph17061909

**Published:** 2020-03-15

**Authors:** Patricia Pendry, Alexa M. Carr, Nancy R. Gee, Jaymie L. Vandagriff

**Affiliations:** 1 Department of Human Development, Washington State University, Pullman, WA 99164, USA; 2 Center for Human-Animal Interaction, Virginia Commonwealth University, Richmond, VA 23298, USA

**Keywords:** university-based animal-assisted intervention, academic skills, risk status

## Abstract

Animal Visitation Programs (AVPs) targeting college students’ stress and academic success have increased, despite limited research on academic outcomes. This randomized controlled trial (*N* = 349) examined the effects of incorporating levels of Human–animal Interaction (HAI) (0%, 50% or 100%) with therapy dogs in a four-week academic stress management program. Conditions included (1) Academic Stress Management (ASM) content only (0% HAI), (2) Human–animal Interaction only (100% HAI) and (3) equal combinations of ASM content and HAI (50% HAI). Intention-to-treat (ITT) analyses examined the effects of students’ risk status (*N* = 146; depression, anxiety, perceived stress, worry) and treatment condition on students’ learning and study strategies at posttest and follow-up. The results showed interactions between condition and risk status demonstrating higher posttest levels of WILL (i.e., anxiety, attitude, motivation) (*Β* = 0.582, *p* = 0.005) and SELFREGULATION (i.e., concentration, self-testing, study aids, time management) (*Β* = 0.501, *p* = 0.031) for at-risk students receiving equal combinations of HAI and content presentations. Moderation effects remained at follow-up (*Β* = 0.626, *p* = 0.005; *Β* = 0.630, *p* = 0.007). At-risk students receiving only HAI (100%) also showed higher levels of WILL at posttest (*Β* = 0.481, *p* = 0.021) and follow up (*Β* = 0.490, *p* = 0.038). University administrators should consider providing at-risk students with targeted programs with varying levels of HAI and ASM content, depending on the targeted academic outcome.

## 1. Introduction

College students are reporting high levels of stress, anxiety, and depression each year [1,2,3], which, according to some researchers, college administrators, and the popular media, constitutes a global ‘college student mental health crisis’ (e.g., [4,5,6,7]). Those concerns are informed by a wide variety of sources including reports of campus-based mental health service utilization [8,9], surveys of counseling center directors (e.g., [10]), and large-scale annual inventories of students’ self-reported distress, psychiatric diagnoses and treatment, and academic impairment (e.g., [1,2]). These reports share similar observations: students are seeking treatment at increasing rates [2,8] for more severe psychological problems [10], and of the small portion (12 to 15%) of students who complete their college’s annual health survey (e.g., [1]), many report ‘more than average’ (45.1%) or ‘tremendous’ (12.7%) stress in the previous year [1] (p. 39), while the majority report feeling ‘overwhelmed by all you had to do’ just in the last 2 weeks (54.7%). A substantial number of students indicate academics (48.2%), finances (31.3%), sleep difficulties (30.9%), intimate relationships (29.6%), and family problems (28.3%) to be ‘traumatic or very difficult to handle’ [1] (p. 36–37). In another survey of over 60,000 college students randomly sampled from 60 U.S. institutions [3] (p. 3), about a third of college students screened positive for depression (37%) and generalized anxiety disorder (31%) using measures of anxiety and depressive symptomology common to medical and clinical settings (PHQ-9 [11]; GAD-7 [12]). The high prevalence of mental health issues is a serious problem, as the presence of stress-related symptoms and disorders decreases students’ performance and increases risk of dropout [13,14,15].

While the developmental pathways underlying links between stress exposure and academic success and failure are complex, there is evidence to suggest that the physiological consequences of academic stress exposure directly compromise students’ motivation and attitudes towards learning [16]. In addition, the lack of academic stress management skills is thought to play a role [17]. High levels of perceived distress in the face of inadequate coping resources leave individuals at risk of developing larger psychiatric problems, especially when this distress is prolonged or severe [17,18,19,20]; the emphasis on perception is key, as this is integral to one’s physiological and emotional response to stress [18,21]. Although some suggest that secondary and higher education institutions should implement structural reforms to reduce academic stress exposure, calls for investment in mental health education and prevention have increased significantly (e.g., [2,22]). Since academic stress is considered an inevitable part of college life, university administrators are eager to identify effective programs that strengthen students’ coping skills, prevent physiological dysregulation, and promote adaptive learning attitudes, skills and strategies in the context of high-stress college life. 

### 1.1. Campus-Based Animal Assisted Interventions

One stress management approach that has been enthusiastically received by university administrators and students is the use of Animal Assisted Intervention (AAIs). There are a variety of types of AAIs, which are defined by the International Association of Human-Animal Interaction Organizations (IAHAIO), a global association of organizations that engage in practice, research, and/or education in animal-assisted practices worldwide. [2] Depending on the setting and delivery of the AAI, the practice falls into further subcategories. Most relevant for University administrators is the Animal Assisted Activity (AAA), “…a planned and goal-oriented informal interaction or visitation conducted by the human-animal team for motivational, educational and recreational purposes.” Conceptualized as preventive interventions to promote students’ general well-being, nearly 1,000 U.S. College campuses [22,23] conduct AAAs within the context of Animal Visitation Programs (AVPs), which are campus-based visitation programs providing university students with the opportunity to interact with animals on a one-time, drop-in basis. 

### 1.2. Causal Evidence and Gaps in the Literature 

Although the use of true experimental designs, featuring random assignment to conditions, is still limited, evidence is emerging that supports the use of AAAs in college settings. Participating in campus-based AAAs has been shown to positively affect students’ perceived emotion states [23,24], and perceived levels of stress [25,26,27]. Moreover, recent evidence suggests that such effects are not merely perceived; findings showed that 10-minutes of hands-on human–animal interaction (HAI) with shelter dogs and cats significantly reduced students’ cortisol levels, a marker of the Hypothalamic Pituitary Adrenal (HPA) Axis, one of the body’s stress sensitive systems associated with the development of stress-related disorders [28]. 

While promising, there is a significant gap in the literature. In brief, while the effects of occasional AAAs on students’ affective and physiological outcomes are encouraging, virtually nothing is known about their effects on students’ academic outcomes. In addition, little is known about the role of dosage, and whether more frequent or intense engagement with animals provides cumulative benefits, and if so, in which domains of functioning. In addition, no studies have examined whether regular exposure to college-based AAAs provides benefits for college students over and above the effects expected from exposure to more traditional, evidence-based stress management approaches. Last but not least, we know little about for whom exposure to HAI is most effective. For example, while mostly implemented as a universal stress prevention tool, we know little about the efficacy of AAAs to reduce stress in college students. This is unfortunate, as most college-based AAAs are expected to contain a mixture of individuals with varying levels of functioning and well-being [29]. Given that there is evidence to suggest that individuals diagnosed with mood disorders may differentially respond to interventions targeting stress-related symptoms [30,31], it is important to understand whether the presence of stress-related symptoms moderates the effects of AAAs on targeted outcomes.

### 1.3. Moderation of Risk Status 

Few studies have examined the moderating effects of students’ mood-related risk status on the efficacy of college-based AAAs. Exceptions are results by Pendry, Vandagriff, and Carr [32], who showed that clinically depressed students reported significantly higher levels of momentary negative emotion including irritability, depression, and anxiety after waiting in line for their turn to engage in hands-on petting of shelter cats and dogs compared to nondepressed students. Similarly, recent findings showed that students who had experienced a mental health problem, learning disability, suicidal ideation, or academic failure experienced significant improvement in aspects of Executive Functioning (EF) after attending four weekly, hour-long sessions focused on interacting with therapy dogs and their handlers, whereas students without such risk factors or those who received traditional evidence-based stress management lectures did not experience such improvements. While the potentially underlying mechanisms were not examined, results reflect the notion that EF can be improved by programs that help individuals feel calm, socially supported, or that enhance emotional well-being [33]. It is thus possible that EF is an aspect of cognitive functioning that is uniquely sensitive to exposure to HAI by reducing students’ perception of stress and anxiety. Whether exposure to HAI exerts similar effects on the cognitive skills associated with academic success is unknown, and therefore, is the focus of this study. 

### 1.4. The Current Study: Effects of HAI on Student Learning Skills and Study Strategies 

The main goal of the current study was to conduct a randomized controlled trial to determine whether, under which conditions, and for whom, a university-based animal assisted stress prevention program effectively promoted students’ learning skills and study strategies. Learning skills and study strategies were examined as they capture aspects of engagement in educationally purposeful activities, skills and competencies, persistence, acquisition of desired knowledge, attainment of educational outcomes, which are synthesized as defining student success [34](p.5). To accomplish this, we examine whether exposure to HAI, evidence-based academic stress management content, or an equal combination of both impacts students’ learning skills and study strategies using a standardized measure. We thus randomly assigned a group of undergraduate students to one of three four-week long programs featuring varying levels of exposure to AAAs and evidence-based stress management content. To examine for whom incorporating AAAs may be most beneficial, we also examined the moderating effects of higher than average levels of stress-related symptoms including depression, anxiety, worry, and perceived stress. We examined treatment effects immediately following completion of the 4-week program, and again 6 weeks after program completion at follow up. 

## 2. Materials and Methods 

This study was conducted at Washington State University in Pullman, WA. After piloting all procedures during the Spring semester of 2016, data collection commenced in the Fall semester of 2016 lasting through the Spring semester of 2018 over a four-semester period. All protocols were approved by the Institutional Animal Care and Use Committee (IACUC #04785-006), the WALTHAM™ Animal Welfare and Ethical Review Board, and the university’s Institutional Review Board (IRB #14918-005). Informed consent was obtained in-person by the Principal Investigator. 

### 2.1. Recruitment 

The study team extended invitations to study informational sessions by approaching undergraduate students through announcements in classes in a wide variety of majors, university publications, and through university-based counselors. Participants viewed a slide presentation describing the study procedures and interested participants were asked to consider their availability to attend the complete series of assessments and program sessions at dates indicated on their screening survey. Students were told that the purpose of the study was to examine the effects of incorporating HAI into existing stress prevention programs offered at the University. Screening surveys contained a ‘blind’ condition indicator based on a combination of a numeric and alphabetical code generated using a simple random sampling method. 

To facilitate the recruitment of a sample containing ample number of students at-risk of academic failure, students completed a screening survey asking students to endorse indices of risk, including being formerly or presently declared academically deficient, diagnosed with a mental condition or disorder, considered suicide or self-harm, and/or receiving classroom accommodations for learning disorder(s). Used as an inclusion criteria, we invited participants (*N* = 349) based on our goal of recruiting an adequately powered sample with balanced representation by condition of typical and at-risk participants, as well as inclusion of both genders. Exclusion criteria included being younger than age 18, reporting a history of hurting animals within the past 3 years, and participation in an academic stress management workshop within six months of study participation.

**Figure 1:**
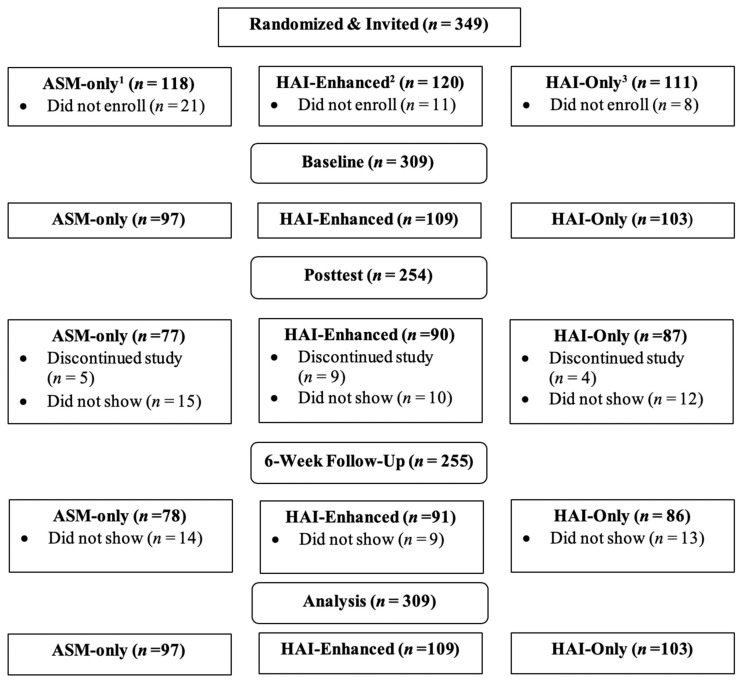
Flow Diagram describing sample randomization, program participation and study completion. ^1^ Academic Stress Management (100% content; 0% HAI); ^2^ Human-Animal Interaction—Enhanced (50% content; 50% HAI); ^3^ Human-Animal Interaction only (0% content; 100% HAI). See 2.3 for more information.

The study was conducted over four semesters during which four cohorts were recruited using identical procedures. Participants who enrolled (*N* = 309) were primarily white (*n* = 78.6%), female (*n* = 243), freshman (*N*_freshman_ = 160, *N*_sophomore_ = 67, *N*_junior_ = 42, *N*_senior_ = 19, *N*_unknown_ = 21), *M_age_ =* 19 years, and taking an average of 15.2 credits. Participation spanned a period of 12 weeks, with the first week spent completing baseline assessments (Week 1), followed by participation in a series of four consecutive weeks of one-hour long programming sessions (Week 2–5), followed by a week of posttest assessments (Week 6), a hiatus of six weeks, and then follow-up assessments (Week 12). Participants included in the current analyses are those who attended the baseline assessment and at least one program session. A flowchart describing enrollment, random assignment and completion of study procedures and assessments is provided in Figure 1. 

### 2.2. Measures

#### 2.2.1. Dependent Variable: Learning and Study Strategies 

The Learning and Study Strategies Inventory 2nd Edition (LASSI [35]) is a diagnostic and prescriptive measure that assesses three main components of strategic learning among college students. It provides a diagnosis of students’ strengths and weaknesses, compared to other college students, and it is prescriptive in that it provides feedback about areas where students may be weak and need to improve their knowledge, skills, attitudes, motivations, and beliefs. The measure consists of an 80-item participant survey comprised of 10 eight-item subscales scored on a 5-point Likert scale (1—*not at all typical* to 5—*very much typical*), resulting in three composite scales referred to as *Will, Skill,* and *Selfregulation*. LASSI demonstrates good reliability as measured by Cronbach Alpha of 0.73–0.89 [35]. Although the LASSI surveys provide self-scoring instructions to survey takers, participants were instructed to ignore scoring instructions and instead to focus only on answering the survey questions. The study team subsequently created and executed syntax reflecting LASSI scoring instructions utilizing reverse scoring for indicated items when calculating subscales. Subscales were standardized and averaged to create their associated composite scores. The LASSI was completed at baseline, posttest and follow-up. 

The first composite scale is *WILL,* which assesses the degree to which students worry about their academic performance, their receptivity to learning new information, their attitudes and level of interest in college, their diligence, self-discipline, and willingness to exert the effort necessary to successfully complete academic requirements. Subscales included are *anxiety* (ANX: reverse-scored; worry about school and academic performance), *attitude* (ATT: attitude and interest in college and achieving academic success), and *motivation* (MOT: diligence, self-discipline, and willingness to exert the effort necessary to successfully complete academic requirements). 

The second composite scale is *SELFREGULATION*, which measures how students manage, or self-regulate and control, the whole learning process through using their time effectively; focusing their attention and maintaining their concentration over time; checking to see if they have met the learning demands for a class, an assignment or a test; and using study supports such as review sessions, tutors or special features of a textbook. Subscales include *concentration* (CON: ability to direct and maintain attention on academic tasks), *self-testing* (SFT: reviewing and comprehension monitoring techniques), *study aids* (STA: use of support techniques, materials and resources to support learning and recalling new information), *time management* (TMT: application of time management principles for academic situations). 

The third composite scale constitutes the *SKILL* component of students’ use of learning strategies which refers to learning strategies, skills, and thought processes related to identifying, acquiring and constructing meaning for important new information, ideas, and procedures, and how they prepare for and demonstrate their new knowledge on tests or other evaluative procedures. Subscales include aspects of *selecting main ideas* (SMI: identifying important information for further study from less important information or supporting details), *information processing* (INP: use of learning strategies like imagery, verbal elaboration, and reasoning skills to help learn new information and connect what is known to what they are trying to learn), and *test strategies* (TST: use of both test preparation and test taking strategies). LASSI subscale scores were standardized for analyses. 

#### 2.2.2. Moderating Variable: Mood Risk Indicator

Given our interest in examining whether the program effects were moderated by students existing stress-related symptoms at baseline, we calculated a mood risk indicator variable based on the presence of symptomology of four mood disorders prevalent in university student populations, i.e., depression, anxiety, worry, and perceived stress. Depression was measured using the 21-item Beck Depression Inventory [36], a reliable measure among young adults (α > 0.83) examining symptoms (i.e., I cry all the time) and attitudes (i.e., I feel sad) frequently expressed by clinically depressed individuals. In our sample, 17.2% endorsed moderate or greater levels of depression, with 6.8% experiencing severe depression. Anxiety was measured using the 21-item Beck Anxiety Inventory [37], a reliable questionnaire (α = 0.88; [38]) assessing the presence of common symptoms of anxiety (i.e., fear of the worst happening, numbness and tingling). In our sample, 19.8% of the sample endorsed moderate or greater levels of anxiety, with 3.9% experiencing levels of anxiety with potential cause for concern. Worry was assessed using the Penn State Worry Questionnaire [39]. This 16-item scale, which has been demonstrated to be reliable among university students (α = 0.83–0.93; [40]), assessed the frequency of (i.e., I worry all the time) and ability to control (i.e., my worries overwhelm me) worry. Perceived-stress was measured using the 10-item Perceived Stress Scale [41] and assessed the extent to which events during the month were stressful (i.e., how often have you felt nervous or stressed). Using each measure’s scoring guidelines, indicator variables (0 = below the mean; 1 = above the mean) for each measure were averaged, resulting in the assignment of an indicator variable for mood risk for each participant (*N_1_* = 146). 

### 2.3. Treatment Conditions

During the screening process, students were randomly assigned to one of three conditions that varied in the ratio of exposure to evidence-based content on academic stress management and human–animal interaction. Students randomly assigned to the *Academic Stress Management condition* (ASM) engaged in an existing, evidence-based program using content presentations (e.g., slide presentations featuring evidence-based information by a masters level health educator), guided activities focused on enhancing self-regulation (e.g., progressive muscle relaxation, deep breathing, meditation, replacing negative self-talk with positive self-talk), and metacognitive skill training (e.g., time management, test taking skills, study planning, goal setting, prioritization exercises). This program condition was conceptualized as a typical form of treatment, i.e., which did not feature any exposure to animals or animal assisted activities (*N* = 97; *N_risk_* = 44; 0% HAI). 

Students assigned to the *Human–Animal Interaction condition* (HAI-O) featured semi-structured HAI sessions during which students engaged in animal assisted activities (e.g., petting/stroking the dog, relaxation activities, meditation, discussion with peers) with therapy dogs and their handlers for the entire program period without any exposure to evidence-based ASM content (*N* = 103; *N_risk_* = 52; 100% HAI). 

Students assigned to the *Enhanced Human–animal Interaction condition* (HAI-E) divided their time equally, within each session, between engaging in a modified ASM curriculum using the same evidence-based content and activities described above (e.g., self-regulation, metacognitive skill development) and exposure to animal assisted activities conducted in the HAI-O condition, during which students interacted with therapy dogs and their handlers (*N* = 109; *N_risk_* = 50; 50% HAI). 

Assessments and sessions were conducted on separate, consistent days by condition using the same health educator, facilitators, research staff and therapy dog teams across conditions. To avoid condition-specific attrition, all participants, including those in the ASM condition, were told they would experience an opportunity to interact with animals, but that the *timing and amount* of HAI would vary by condition, as such blinding them to the expected ratio of exposure to HAI versus content as much as possible. For ethical reasons, exposure to HAI was provided to the ASM group after completion of outcome assessments. All participants were compensated up to $60 USD for completing assessments, which were prorated at $20 USD per assessment.

#### Human–Animal Interaction

Participants in the HAI conditions interacted with registered therapy handler-dog teams who were trained and evaluated members of a local regional community partner of the Pet Partners national organization [42]. On any given program day, the total number of handler-dog teams varied between 5–7, depending on the total number of participants, to achieve a dog:student ratio of 1:4. Teams consisted of 16 male dogs (15 neutered, 1 intact) and 11 female dogs (8 spayed; 3 intact) (*M*_age_ = 4 years, Age_Max_ = 12 years, Age_Min_ = 6 months). The majority of dogs were Labrador Retrievers (*n* = 10), mixed breeds (*n* = 6), and Golden Retrievers (*n* = 3) (*n_other_* = 8). On average, dogs had been registered with Pet Partners for 1.95 years upon study commencement (range: 1 month– 6 years) and participated in 3.6 hours of therapy work per week (range: 1 hour–15 hours per week). The majority of handlers were female (*N* = 24; M_age_ = 49.67; Age_Min_ = 26, Age_Max_ = 70), with 2.34 years of AAA experience (range: 1 month–6 years). During HAI sessions, each handler-dog team was assigned to one of seven segmented sitting areas where handler-teams sat on couches or on the floor for the duration of the session. Each handler-dog team attended an orientation meeting prior to participating, in which the study and facilitation procedures described below were explained in detail. Dogs postural state was allowed to occur based on their and their handler’s preference, although teams were encouraged to stay within the assigned seating area. 

### 2.4. Description of Session Outlines, Activities, and Themes

All program sessions, including those not featuring HAI, occurred in the same carpeted conference room in a building located at the center of campus, which featured a large center table and perimeter sofas, chairs, and side-tables arranged to form seven segmented sitting areas. Each weekly session featured a central theme related to promoting academic success including academic stress management, motivation and goal setting, benefits of sleep, and test anxiety. Students arrived between 5–30 min before program sessions started and were checked in outside the interaction space by the PI and graduate students. Students waited out of view of the animals before entering the room, which occurred as a group at a predetermined start time. Upon entry, groups of 4–5 students were encouraged to approach a handler-dog team of their choosing and to do so while minimizing crowding of the animals. Participants in the ASM condition were directed to seat themselves at a large center table.

Regardless of the theme featured that week, program activities were sequenced in the following manner. Students spent the first 20 min of the program in various combinations of receiving evidence-based content through slide presentations by the same Master level mental health and promotion specialist (ASM) and/or meeting and/or greeting their peers (ASM) or handler-dog teams (HAI-E/HAI-O). Students assigned to the ASM group received 20 min of content presentations only, those in the HAI-E condition received a combination of 10 min of meet and greet focused HAI and 10 min of content presentations. Participants in the HAI-O group did not receive any evidence-based content presentations, but engaged in 20 min of meet-and-greet focused HAI. For the remainder of the session, all participants, regardless of condition, engaged in two 10-minute-long guided activities: one focused on mindfulness, meditation, relaxation, or visualization, and the other on small-group, semi-structured discussions and reflections. The order of the activities alternated weekly, and depending on students’ assigned treatment condition, the facilitation scripts were modified. For participants in groups receiving content (ASM and HAI-E), scripts referred to terminology reflecting information shared during content presentations (i.e., “think about a *process-oriented* goal”), whereas terminology for the HAI-O group was modified to reflect more general speech (i.e., “think about a *reasonable* goal”). Additionally, for students assigned to the HAI-O and HAI-E conditions, guided activities included explicit instructions to touch and stroke the dogs throughout the guided activities. At the end of each session, students in each condition engaged in discussion with peers; those in HAI-O and HAI-E conditions did so in the presence of the dogs. 

#### 2.4.1. Session 1: Academic Stress Management

Content presentations for students in the ASM and HAI-E conditions focused on manifestations of stress and effective self-care practices to manage stress. Next, participants in each condition were guided through a breathing and body scan exercise. For the HAI conditions, this exercise was conducted while sitting and petting and touching the dogs and receiving instruction on ‘experiencing’ the dog they were with. Finally, each condition engaged in a discussion activity during which participants sat in small groups with their peers and/or handler-dog teams (HAI-E; HAI-O) in the segmented sitting areas. The discussion activity in the ASM and HAI-E conditions was semi-structured, guided by prompts using terms introduced during the content presentations, focused on identifying and reframing current stressors, as well as discussing students’ use of coping strategies. The HAI-O group engaged in a similar but less structured discussion, using general prompts and terms on the same topic. Throughout the discussion activity, participants in the HAI conditions were encouraged to pet and interact with the handler-dog teams.

#### 2.4.2. Session 2: Motivation and Goal Setting

Content presentation for students in the ASM and HAI-E conditions focused on identifying and setting attainable goals, establishing behavioral habits to support their completion, including enhancing a growth rather than fixed-mindset, and engaging in self-talk towards goal completion. Next, participants in each condition were guided through a discussion activity during which participants sat in small groups with their peers and/or handler-dog teams (HAI-E; HAI-O) in the segmented sitting areas. The discussion activity in the ASM and HAI-E conditions was semi-structured, guided by prompts using terms introduced during the content presentations, focused on setting attainable academic goals, addressing the anticipated steps necessary, and identifying behavioral modifications towards goals completion. The HAI-O group engaged in a similar but less structured discussion, using general prompts focused on identifying a reasonable academic goal for the semester, why that goal was meaningful, and what barriers they may encounter in achieving it. Throughout the discussion, participants in the HAI conditions were encouraged to pet and interact with the handler-dog teams. Lastly, participants in each condition completed a visualization exercise, during which they were encouraged to witness themselves going through the steps they explored during discussion, concluding with successful completion. For HAI conditions, this exercise was conducted while sitting and petting the dogs. 

#### 2.4.3. Session 3: Benefits of Sleep

Students in the ASM and HAI-E groups received information on the amount of healthy sleep needed, the effects of sleep deprivation, and how to overcome common barriers through instruction on identifying behavioral modifications focused on creating optimal sleep environments, routines, and behavior. Next, all participants were guided through a progressive muscle relaxation meditation to provide practice at deliberate relaxation to be used as part of a bedtime routine. For HAI conditions, this exercise was conducted while sitting with and touching the dogs. Next, participants in each condition were guided through a discussion activity during which participants sat in small groups with their peers and/or handler-dog teams (HAI-E; HAI-O) in the segmented sitting areas. A similar, semi-structured discussion format using similar prompts was used for each condition focused on exploring the quality of students’ current sleep environments and actions they would be willing to take to improve their sleep environment. For HAI conditions, this exercise was conducted while sitting and petting the dogs. 

#### 2.4.4. Session 4: Test Anxiety

Students in the ASM and HAI-E conditions received information about what anxiety is, how it can manifest physically and mentally, and approaches to overcoming it. Next, using identical prompts for each condition, all participants engaged in a visualization activity. For students in ASM and HAI-E groups, this visualization was conducted around the central table, while students in the HAI-O group sat in segmented areas with the dogs. The first 5 minutes of the activity was intended to evoke feelings of stress and anxiety about a fictional but upcoming exam. The next 10 minutes consisted of a stress-release meditation, which included techniques to interrupt disruptive thoughts and feelings, encourage a calm state, and visualize successfully completing the fictional exam. For groups in the HAI conditions, the stress-release activity was conducted in the presence of animals and prompted students to think about the dog’s calming presence. Lastly, all students engaged in a discussion activity which focused on reflecting on their experience with the prior activity. They also reflected on how they could utilize the skills practiced in the mindfulness activities throughout the four program weeks to manage and/or interrupt experiences of stress and anxiety after program completion.

### 2.5. Program Fidelity

Program sessions were highly structured and controlled, featuring strict adherence to lecture notes on content by the same trained facilitation staff and experienced health educator who facilitated consistently across conditions. Curriculum content was presented with the aid of detailed, memorized scripts including meditation and relaxation activities. Facilitators adhered to sequences of 30-second intervals, which were monitored and prompted by a research assistant who kept a record to document the fidelity of presented content and facilitation of activities. All sessions featuring HAI were video-recorded from seven different simultaneous camera angles.

### 2.6. Power Calculation and Sample Size

Given that Cohen’s *d* effect sizes ranged from as small as 0.2 to as large as 1.2 in animal assisted intervention work [43], we plotted the necessary sample size to achieve adequate power over a range of Cohen’s *f*^2^ effect sizes from 0.1 through 0.5, corresponding to Cohen’s *d* values ranging from 0.2 through 1.2. Given the three-group structure of our study and our interest in exploring interactions by risk-status (a 2x3 factorial design) with at least a single covariate, the total sample size ranged from a low of 100 to a high of 210 to maintain power of 0.80. This indicated that per condition, 33 to 70 students would be required to have adequate power to detect an effect size as low as 0.2 with power of 0.8. 

### 2.7. Analytic Approach: Moderation of Treatment Effects by Levels of HAI and Risk Status 

Multivariate regression analyses were used to answers two research questions. The first research question focused on understanding whether or not there were differences in students’ levels of the three subscales of learning and study strategies (i.e., will, selfregulation and skill) by risk status (0,1) and treatment condition (e.g., ASM, HAI-E, HAI-O) after program completion (posttest). The second question examined whether those differences by group and condition were present 6 weeks later (at follow-up, assessed at 12 weeks). To reduce potential bias in treatment effects arising from missing outcome data, we conducted an intention-to-treat (ITT) analysis to test for interactions between student’s treatment conditions by risk status on learning and study strategies, first at posttest, and then again by running the same model predicting learning and study strategies at follow-up. Using indicator variables for each condition, e.g., whether in the ASM condition (0,1), whether in the HAI-E condition (0,1), whether in the HAI-O condition (0,1), whether identified at-risk (0,1) and interaction terms created by multiplying indicators for each condition with risk status, we modeled the contributions of the main and interaction effects of the presence of general risk by treatment condition. Even though there was no indication that attrition occurred systematically by condition, we controlled for the total number of sessions that participants attended. The results were interpreted with the high-risk students in the ASM condition serving as the reference category. Based on pooled estimates, we interpret contributions of interaction effects of the presence of mood risk and treatment condition for all three aspects of learning and study strategies at posttest and then, using identical models, at follow-up. 

## 3. Results

### 3.1. Differences at Baseline by Condition and Risk Status

Analyses were conducted using IBM SPSS Statistics for Windows, version 26. An examination of distribution across the three treatment conditions revealed that *mood risk, X*^2^ (2, *N* = 291) = 0.293, *p* = 0.864, class standing, *X*^2^(10, *N* = 274) = 6.97, *p* = 0.728, race, *X*^2^(12, *N* = 277) = 7.65, *p* = 0.812, and gender, *X*^2^(2, *N* = 277) = 6.38, *p* = 0.896, were equally distributed across conditions. Using a one-way ANOVA, the results also showed no differences by condition in students’ age *F*_(2,273)_ = 2.08, *p* = 0.127. Additionally, there were no statistically significant differences by condition at baseline among the composite factors of the main dependent variable of interest, nor their respective subscales: WILL, *F*_(2,280)_=0.72, *p* = 0.49, *anxiety* (ANX), *F*_(2,279)_ = 2.41, *p* = 0.09, *attention* (ATT), *F*_(2,279)_ = 0.080, *p* = 0.92, *motivation* (MOT), *F*_(2,278)_ = 0.331, *p* = 0.72; SELFREGULATION, *F*_(2,279)_ = 0.461, *p* = 0.63, *concentration* (CON), *F*_(2,277)_ = 0.769, *p* = 0.47, *self-testing* (SFT), *F*_(2,278)_ = 0.491, *p* = 0.61, *study aids* (STA), *F*_(2,278)_ = 0.726, *p* = 0.49, *time management* (TMT), *F*_(2,279)_ = 0.759, *p* = 0.47; and SKILL, *F*_(2,279)_ = 1.40, *p* = 0.25, *information processing* (INP), *F*_(2,278)_ = 1.53, *p* = 0.22, *selecting main ideas* (SMI), *F*_(2,278)_ = 1.69, *p* = 0.19, *test strategies* (TST), *F*_(2,278)_ = 0.384, *p* = 0.68. Further examination of differences at baseline in the main dependent variables of interest, and their respective subscales by *mood risk and condition* indicated that mean levels on composite scores were similar across treatment conditions for WILL, *F*_(2,148)_ = 2.34, *p* = 0.100, SELFREGULATION, *F*_(2,147)_ = 1.49, *p* = 0.229, or SKILL, *F*_(2,147)_ = 2.85, *p* = 0.061 for typical students (*mood risk = 0*). Likewise, at-risk students (*mood risk* = 1) showed similar levels across conditions for WILL, *F*_(2,129)_ = 0.677, *p* = 0.510, SELFREGULATION, *F*_(2,129)_ = 2.13, *p* = 0.123, or SKILL, *F*_(2,129)_ = 0.371, *p* = 0.690 at baseline. As expected, at baseline, LASSI scores for students identified as at-risk were lower compared to those identified without the presence of mood related symptoms on WILL, *F*_(1, 281)_ = 47.45, *p* < 0.001, SELFREGULATION, *F*_(1, 281)_ = 47.45, *p* < 0.001, and SKILL, *F*_(1, 281)_ = 47.45, *p* < 0.001. Last, total attendance was equally distributed across condition, *X*^2^(8, *N* = 309) = 6.36, *p* = 0.607 and mood risk *X*^2^(4, *N* = 309) = 4.515, *p* = 0.341. 

### 3.2. Predicting Effects of Treatment Condition and Mood Risk Status on WILL

Model statistics reveal a significant interaction between the presence of mood risk and treatment condition on students’ scores on WILL, R^2^ = 0.197, *F*_(6,302)_ = 12.31, *p* < 0.001. Pooled results suggest that at-risk students assigned to the HAI-E condition (B = 0.582, *p* = 0.005) and HAI-O condition (B = 0.481, *p* = 0.021) showed significantly higher levels of WILL at posttest than those at-risk assigned to the ASM condition, i.e., the reference group. These findings are independent of students’ level of attendance to the program, which was not associated with the dependent variable WILL at posttest, B = 0.066, *p* = 0.231. Regression analyses predicting WILL scores at the six-week follow-up also support a significant interaction between presence of mood risk and treatment condition on students’ scores on WILL, R^2^ = 0.126, *F*_(6,302)_ = 7.26, *p* < 0.001. Results show that high-risk students assigned to the HAI-E condition (B = 0.626, *p* = 0.005) and HAI-O condition (B = 0.490, *p* = 0.038) demonstrated significantly higher levels of WILL at follow-up than those at-risk assigned to the ASM condition. These findings are independent of students’ level of attendance to the program, which was not significantly associated with the dependent variable at follow up (B = 0.073, *p* = 0.091).

**Figure 2:**
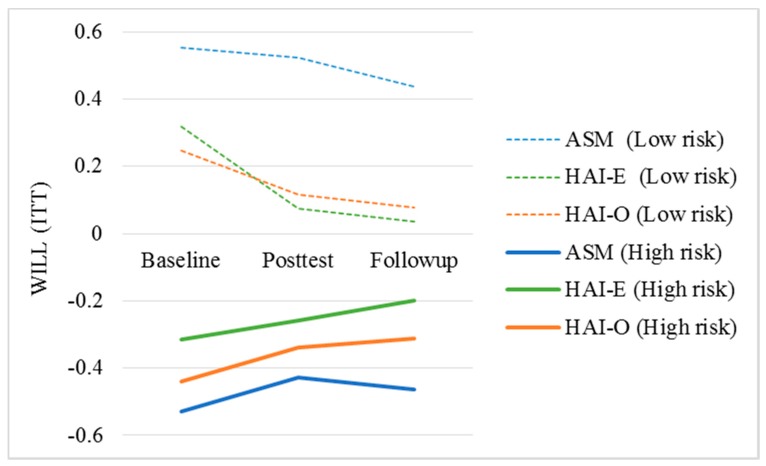
Trajectory of WILL composite scores by treatment condition and risk status. ASM: Academic Stress Management; HAI-E: Human–Animal Interaction Enhanced; HAI-O: Human-Animal Interaction only; ITT: Intention-to-treat.

Results (Table 1) are depicted in Figure 2 showing mean levels of WILL by risk status and condition at three time points, at baseline, posttest, and follow-up. Mean scores at posttest and follow up are presented by risk status and condition based on predicted scores generated by the regression models at both time points. As expected, the results also clearly show significant differences in students’ composite scores of WILL by risk status, suggesting that at-risk students had lower levels of learning and study strategy functioning at posttest (B = −0.934, *p* < 0.001) and at follow-up six weeks later (B = −0.880, *p* < 0.001) compared to students with below average levels of anxiety, depression, worry, and perceived stress. In sum, compared to at-risk students who exclusively received evidence-based academic stress management content, at-risk students who interacted with therapy dogs, exclusively or in combination with content exposure, had significantly higher scores of WILL after the intervention, which remained 6 weeks later.

### 3.3. Effects of Treatment Condition and Mood Risk Status on SELFREGULATION 

Model fit statistics indicate the presence of a significant interaction between presence of risk and treatment condition on students’ self-regulation scores at posttest R^2^ = 0.084, *F*_(6,302)_ = 4.60, *p* < 0.001. Results show that at-risk students assigned to the HAI-E condition (B = 0.501, *p* = 0.031) experienced significantly higher levels of SELFREGULATION at posttest than at-risk students assigned to the ASM condition. No interaction effects were found for students assigned to the HAI-O condition, (B = 0.268, *p* = 0.215) at posttest. These findings are independent of students’ level of attendance to the program, which was not significantly associated with the dependent variable (B = −0.006, *p* = 0.931) at posttest.

At follow up, using the same set of predictors, model fit statistics indicate the presence of a significant interaction between the mood risk and treatment condition on students’ scores on SELFREGULATION, R^2^ = 0.088, *F*_(6,302)_ = 4.84, *p* < 0.001. The results suggest that at-risk students assigned to the HAI-E condition (B = 0.630, *p* = 0.007) experienced significantly higher levels of academic-related self-regulation at follow-up than at-risk students assigned to the ASM condition. No interaction effects were found for students assigned to the HAI-O condition at follow up (B = 0.364, *p* = 0.148) as compared to at-risk students in the ASM condition. These findings are independent of students’ level of attendance to the program which was not significantly associated with the dependent variable at follow up (B = 0.041, *p* = 0.539). In sum, at-risk students who interacted with therapy dogs in combination with content exposure had significantly higher scores of SELFREGULATION after the intervention that remained 6 weeks later.

**Figure 3:**
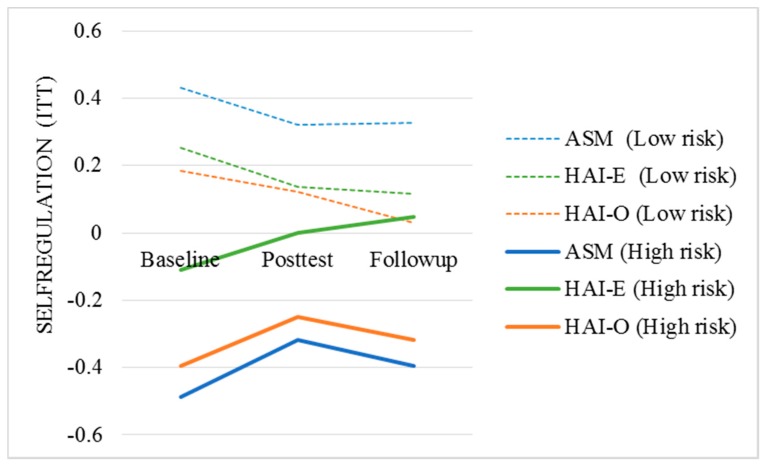
Trajectory of SELFREGULATION composite scores by treatment condition and risk status. ASM: Academic Stress Management; HAI-E: Human–Animal Interaction Enhanced; HAI-O: Human-Animal Interaction only; ITT: Intention-to-treat.

Results (Table 2) are depicted in Figure 3 showing mean levels of SELFREGULATION by risk status and condition at baseline, posttest, and follow-up based on predicted scores generated by the regression models. As expected, students with higher than average mood symptoms had lower levels of learning and study strategy functioning (B = −0.638, *p* < 0.001) at posttest and again at 6-week follow-up (B = −0.710, *p* < 0.001) compared to students without mood symptoms. 

### 3.4. Effects of Treatment Condition and Mood Risk Status on SKILL 

**Figure 4:**
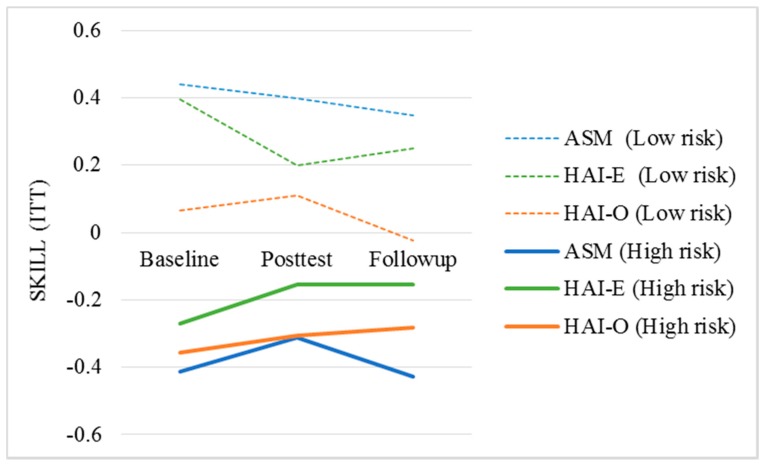
Trajectory of SKILL composite scores by treatment condition and risk status. ASM: Academic Stress Management; HAI-E: Human–Animal Interaction Enhanced; HAI-O: Human-Animal Interaction only; ITT: Intention-to-treat.

Model fit statistics (R^2^ = 0.116, *F*_(6,302)_ = 6.63, *p* < 0.001) indicate the presence of significant interaction between the mood risk and treatment condition on students’ scores on SKILL. The results (Table 3 and Figure 4) show significant differences in students’ composite scores of SKILL, R^2^ = 0.116, *F*_(6,302)_ = 6.63, *p* < 0.001, by risk status, suggesting that at-risk students have lower levels of learning and study strategy functioning, B = −0.704, *p* < 0.001, at posttest and again at follow up (B = −0.756, *p* < 0.001). However, we found no significant interaction effects by risk-status and the HAI-E condition at posttest (B = 0.340, *p* = 0.176), or follow up (B = 0.333, *p* = 0.149) on SKILL. Similarly, no significant interactions were found for risk-status and participation in the HAI-O condition at posttest, (B = 0.288, *p* = 0.227). At follow up (R^2^ = 0.139, *F*_(6,302)_ = 8.10, *p* < 0.001, results showed a significant interaction among at-risk students assigned to the HAI-O condition (B = 0.499, *p* = 0.029), demonstrating significantly higher use of study skills and strategies compared to the at-risk students in the ASM condition, while controlling for attendance. This suggests that student experiencing higher than average mood symptoms who engaged with therapy dogs without receiving any exposure to content presentations had significantly higher skill levels 6 weeks after the intervention compared to those that received evidence-based content presentations only.

## 4. Discussion

This is the first study to employ a randomized controlled study design to examine whether incorporating human–animal interaction into an evidence-based stress-prevention program can improve behavioral aspects of academic success through an examination of students’ study and learning strategies in typical and at-risk college students. We used a reliable measure of learning and study strategies (LASSI; [35]) to assess three main components of strategic learning (i.e., Will, Selfregulation, and Skill) among university students, who were randomly assigned to one of three conditions involving varying ratios of HAI and formal academic stress management instruction. Students’ scores assessed immediately following the intervention and again 6 weeks later were analyzed by condition while simultaneously considering the moderating influence of students’ risk of academic failure based on the presence of higher than average stress-related mood symptoms including depression, anxiety, perceived stress, and worry. The main findings of this study are that compared to at-risk students who exclusively received evidence-based academic stress management content, at-risk students who interacted with therapy dogs, exclusively or in combination with content exposure, had significantly higher scores of WILL after the intervention, that were also significant 6 weeks later. Similarly, at-risk students who interacted with therapy dogs in combination with content exposure had significantly higher scores of SELFREGULATION after the intervention that remained 6 weeks later. While the interaction effect of risk status and HAI exposure on students’ SKILL was not significantly different immediately following the intervention, the results showed significant interaction effects 6 week later, showing that at-risk students who received HAI but not content exposure had significantly higher skill levels than at-risk students who received instruction only through evidenced based content presentations. Overall, the results provide evidence that interacting with therapy dogs and their handlers, either with or without exposure to formalized stress reduction content, most improves behavioral aspects of academic success for university students who have been classified as being at high risk of academic failure. 

Although this study did not examine the mechanisms that may have mediated the observed effects, there are theoretical models that can help elucidate the observed findings. Although not empirically verified, Gee, Griffin, and McCardle [44] proposed a theoretical framework to explain both the direct effects of HAI on individuals’ motivation, engagement, self-regulation, and social interaction, as well as the indirect effects on learning. In this framework, they explain that HAI may indirectly influence learning by increasing self-regulation and stress coping, or through the promotion of social behaviors, increased calmness, and reduced fear and anxiety. In addition, HAI may directly enhance learning and study skills by enhancing students’ motivation and self-efficacy, or by increasing their engagement and attention. The results from the current study are thus consistent with this framework and extend the effects to university aged students. The significant interaction of risk status and HAI Only exposure on students’ skills six weeks after completion of the intervention is interesting, because the students were encouraged to discuss aspects of academic skills such as goal setting, test taking strategies, and coping with test anxiety during semi-structured discussions with peers while in contact with therapy dogs. The relaxed environment and related discussion may thus have helped stressed students increase their performance on the test-taking strategies subscale. While this result is also consistent with the Gee, Griffin, and McCardle [44] framework, it was an unexpected outcome, and as such, we recommend that future research attempt to replicate and extend this finding. Overall, our results further refine this framework, in that we see a reduction in anxiety and a corresponding increase in motivation and attitudes, as well increased selfregulation and associated behavior, but only for at-risk students, indicating that they probably have different needs from typical students. The lack of a similar finding for lower-risk students is possibly also due to a higher levels of study skills in that group, as well as less impairment in proposed mediating domains (e.g., motivation, self-efficacy etc.) resulting in relatively small improvements that do not meet statistical significance. 

Interestingly, we did not predict the lack of significant improvements in study skills and strategies in response to exposure to evidence-based academic stress management content and activities for typical or at-risk students. Given that the evidence-based content used was specifically designed to reduce academic stress, test anxiety, and sleep problems, while improving motivation and goal setting to enhance predictors of academic success, it is interesting that it did not improve study skills and strategies in our study. With regard to this finding, we can only speculate that it is possible that exposure to ASM content exclusively, i.e., without engagement in HAI, may have had the effect of increasing participant focus on academic challenges which would likely include a corresponding increase in fear, anxiety, and stress. 

While speculative, the overall results resonate with findings from a previously published program evaluation [45] where we examined participants’ responsiveness to animal assisted programming activities. The results of qualitative analyses based on participant responses to open-ended, anonymous survey questions, showed that interacting with a therapy dog may have served as a positive and rewarding stimulus that became associated with the participation in content presentations. Engaging with animals was perceived to be more useful than exposure to the stress prevention content presentations, because the presence of animals created a more positive and calming environment that allowed for optimal onboarding of ASM content and discussion about stressful topics such as academic stress, academic goal setting, and motivation, and discussion about test taking and study strategies. 

### Strengths and Limitations

In addition to randomizing participants to treatment conditions, this study features several strengths in research design and implementation approaches. First, our study is the first to incorporate various levels of HAI and risk status to measure effects of regular, hour-long exposures of HAI over a four-week period on an outcome not previously examined but directly relevant for academic success: college students’ study and learning skills. This is a novel contribution to the literature, as most causal studies examining college-based AVPs have focused on relatively short exposures to HAI and their effects on affective states, perceived stress, and mood. Second, the chosen measure used to assess our main outcome variable, the LASSI, appears to be sensitive to aspects of learning and study strategies that are likely to put those students at-risk of academic failure, as evidenced by the observation that students classified as ‘at-risk’ had lower LASSI scores across all subscales, compared to ‘typical’ students at all time points. Third, not only did this study examine the effects of various levels of HAI, it did so using a robust, real-life comparison condition by comparing effects of various HAI levels to evidence-based stress prevention approaches currently utilized by the university. As such, this study can elucidate not just whether exposure to HAI affects college students functioning and wellbeing, but also whether these effects exist in addition to those experienced through traditional evidence-based stress prevention approaches. Fourth, while the study was conducted in situ during a real-life implementation, exposure to HAI, content or both, was consistent and highly controlled. Facilitators received implementation and curriculum training to ensure high-quality delivery of evidence-based content used in the comparison condition, mimicking real-life, treatment-as-usual conditions. The handlers and animals participating in this study were registered with a national organization whose procedures ensure proper selection, training, and evaluation of qualified handler teams. The use of consistent facilitation staff, scripts, timing, and reviews of video recorded sessions limits the influence of potentially confounding variables due to unintended variation within and between conditions beyond the intended treatment i.e., ratio of HAI and content. As such, we are confident that conditions did indeed provide the ratio of HAI and content as intended without introducing students to third variables or unintended treatments. Fifth, our use of intention-to-treat analyses and multiple imputation reduces the potential bias in estimating treatment effects arising from missing data due to participants having dropped out of the study or having violated the timing of the assessment protocol. Last but not least, our study builds on the existing literature by asking for whom exposure to HAI may be most effective by including both typically-developing students, as well as those at risk of academic failure as indicated by higher than average levels of anxiety, depression, worry, or perceived stress. This feature of our design provides valuable evidence about for whom and under what conditions college-based AVPs are most efficacious. Including low- and high-risk students also generates confidence in the study’s external validity, given that many college-based AVPs provide universal and targeted approaches to students with similar characteristics. 

However, the following limitations must be considered. While we believe this sample is representative of college students who are interested in HAI and likely engage in these types of programs, it may leave out a significant portion of our target population. Individuals who are willing to participate in a study on HAI may have higher level of interest in interaction with animals than students who were approached for participation but decided not to enroll. In addition, we recognize that the unbalanced number of female participants in our sample affects our generalizability. As such, this study speaks mostly to students who are willing to engage in HAI, who may have some belief in the positive effects of such a program, and who are willing to commit to a 4-week long academic stress prevention program featuring weekly sessions, mediation activities, and peer discussion. A second limitation of this study is that the results provide much-needed information about efficacy, but offer little in the way of revealing underlying mechanisms. Third, it is obvious that students’ study skills and strategies are influenced by variables other than those appearing in our models, as is evidenced through our model statistics. As such, it is important to recognize that while the implications of these findings are promising, further research is needed to better understand the underlying causal pathways and the role of highly structured implementation approaches. 

## 5. Conclusions

Overall, the results provide evidence that interacting with therapy dogs and their handlers, but not exposure to formalized stress reduction content, most improves behavioral aspects of academic success for university students who have been classified as being at high risk of academic failure. University administrators should thus consider providing at-risk students with targeted programs with varying levels of HAI and ASM content, depending on academic outcomes targeted.

## Figures and Tables

**Table 1:** Regression analyses modeling moderation effects of mood risk and treatment condition on students’ WILL towards learning and study strategies.

	Posttest			Follow-Up		
	B	SE B	*p*-Value	B	SE B	*p*-Value
(Constant)	0.308	0.205	0.148	0.197	0.184	0.288
HAI-E	−0.439	0.142	0.002 **	−0.390	0.153	0.011 *
HAI-O	−0.406	0.147	0.006 **	−0.356	0.157	0.024 *
Mood Risk	−0.934	0.151	<0.001 ***	−0.880	0.162	<0.001 ***
HAI-E * Mood Risk	0.582	0.209	0.005 **	0.626	0.222	0.005 **
HAI-O * Mood Risk	0.481	0.208	0.021 *	0.490	0.235	0.038 *
Attendance	0.066	0.053	0.231	0.073	0.043	0.091

Note. * *p* < 0.05. ** *p* < 0.01. *** *p* < 0.001. Model statistics presented in-text. Model statistics represent pooled imputation estimates. B: Unstandardized Beta coefficient. SE B: Standard Error for the unstandardized coefficient.

**Table 2:** Regression analyses modeling moderation effects of mood risk and treatment condition on student’s SELFREGULATION of learning and study strategies.

	Posttest			Follow-Up		
	B	SE B	*p*-Value	B	SE B	*p*-Value
(Constant)	0.338	0.233	0.171	0.193	0.255	0.465
HAI-E	−0.183	0.160	0.258	−0.204	0.159	0.200
HAI-O	−0.198	0.148	0.182	−0.297	0.155	0.056
Mood Risk	−0.638	0.160	<0.001 ***	−0.710	0.173	<0.001 ***
HAI-E * Mood Risk	0.501	0.229	0.031 *	0.630	0.233	0.007 **
HAI-O * Mood Risk	0.268	0.215	0.215	0.364	0.249	0.148
Attendance	−0.006	0.065	0.931	0.041	0.064	0.539

Note. * *p* < 0.05. ** *p* < 0.01. *** *p* < 0.001. Model statistics presented in-text. Model statistics represent pooled imputation estimates. B: Unstandardized Beta coefficient. SE B: Standard Error for the unstandardized coefficient.

**Table 3:** Regression analyses modeling moderation effects of mood risk and treatment condition on students’ learning and study strategies SKILL.

	Posttest			Follow-Up		
	B	SE B	*p*-Value	B	SE B	*p*-Value
(Constant)	0.301	0.184	0.105	0.126	0.261	0.640
HAI-E	−0.195	0.171	0.258	−0.089	0.156	0.569
HAI-O	−0.288	0.170	0.092	−0.368	0.157	0.019 *
Mood Risk	−0.704	0.184	<0.001 ***	−0.756	0.170	<0.001 ***
HAI-E * Mood Risk	0.340	0.249	0.176	0.333	0.230	0.149
HAI-O * Mood Risk	0.288	0.238	0.227	0.499	0.228	0.029 *
Attendance	0.031	0.052	0.564	0.068	0.062	0.293

Note. * *p* < 0.05. ** *p* < 0.01. *** *p* < 0.001. Model statistics presented in-text. Model statistics represent pooled imputation estimates. B: Unstandardized Beta coefficient. SE B: Standard Error for the unstandardized coefficient.
